# A Fully 3D-Printed Steerable Instrument for Minimally Invasive Surgery

**DOI:** 10.3390/ma14247910

**Published:** 2021-12-20

**Authors:** Costanza Culmone, Kirsten Lussenburg, Joost Alkemade, Gerwin Smit, Aimée Sakes, Paul Breedveld

**Affiliations:** Bio-Inspired Technology Group (BITE), Department of BioMechanical Engineering, Faculty of Mechanical, Maritime, and Materials Engineering, Delft University of Technology, 2628 CD Delft, The Netherlands; c.culmone@tudelft.nl (C.C.); alkemadejoost@gmail.com (J.A.); G.Smit@tudelft.nl (G.S.); a.sakes@tudelft.nl (A.S.); p.breedveld@tudelft.nl (P.B.)

**Keywords:** additive manufacturing, non-assembly, surgical instruments, ergonomics

## Abstract

In the field of medical instruments, additive manufacturing allows for a drastic reduction in the number of components while improving the functionalities of the final design. In addition, modifications for users’ needs or specific procedures become possible by enabling the production of single customized items. In this work, we present the design of a new fully 3D-printed handheld steerable instrument for laparoscopic surgery, which was mechanically actuated using cables. The pistol-grip handle is based on ergonomic principles and allows for single-hand control of both grasping and omnidirectional steering, while compliant joints and snap-fit connectors enable fast assembly and minimal part count. Additive manufacturing allows for personalization of the handle to each surgeon’s needs by adjusting specific dimensions in the CAD model, which increases the user’s comfort during surgery. Testing showed that the forces on the instrument handle required for steering and grasping were below 15 N, while the grasping force efficiency was calculated to be 10–30%. The instrument combines the advantages of additive manufacturing with regard to personalization and simplified assembly, illustrating a new approach to the design of advanced surgical instruments where the customization for a single procedure or user’s need is a central aspect.

## 1. Introduction

### 1.1. State of the Art

The advent of Minimally Invasive Surgery (MIS) can be considered one of the most important innovations in the surgical field. In MIS, two or three small incisions, usually between 5 and 10 mm in diameter, act as an entry port to the human body, hereby avoiding a large incision, which is common in conventional open surgery. In the small incisions, a temporary port called trocar is used to facilitate the insertion of the instruments. This minimally invasive approach reduces the risk of complications such as infections or hemorrhages, decreases the hospitalization time, and minimizes the size of the scar, reducing the pain for the patient [1,2]. However, different from open surgery where the surgeon has direct visualization and access to the operation area, in MIS, the indirect visualization and the limited operational space to maneuver the instruments influence the surgeon’s performance.

**Figure 1:**
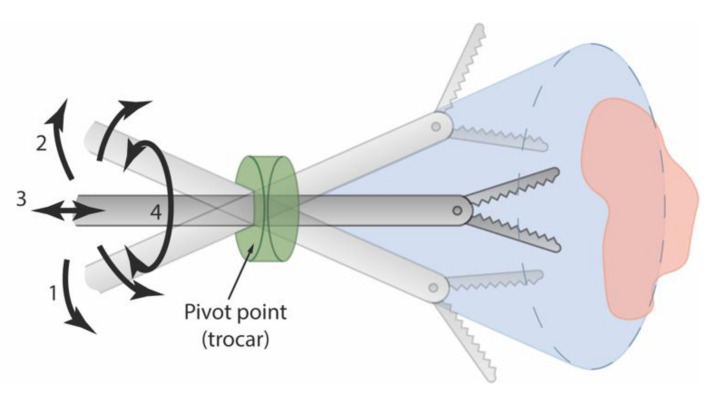
Instrument degrees of freedom in Minimally Invasive Surgery: (1) and (2) pivoting around the incision in two perpendicular planes, (3) axial translation and (4) axial rotation.

Instruments conventionally used in MIS are characterized by three main components: a handle to maneuver the device, a long and straight shaft to reach the operation area, and an end-effector to operate, usually containing a grasper or a cutting mechanism. The rigid and slender instruments used in MIS severely reduce the dexterity of the surgeon due to the loss of wrist articulation and the restriction posed by the small incision size. The number of degrees of freedom (DOF) is limited from six in open surgery to four in MIS (Figure 1): (1) and (2) pivoting on the incision in two perpendicular planes, (3) axial translation, and (4) axial rotation [3]. Aside from the reduced number of DOF, the surgeon has to cope with the fulcrum effect: the inversion of the handle movements at the end-effector due to the pivot point created by the trocar in the abdominal wall.

### 1.2. Challenges in Minimally Invasive Surgery

Solutions have been proposed to overcome the limitations of MIS by enhancing the dexterity of instruments using wrist-like mechanisms. Robotic devices, such as the da Vinci^®^ robotic system (Intuitive Surgical Inc., Sunnyvale, CA, USA), have the ability to diminish the fulcrum effect and enhance the surgeon’s dexterity by providing two additional DOF to the end-effector of the robotic arm using the so-called EndoWrist mechanism. Still, the high initial costs and the limited lifespan of the robotic instruments [4] push researchers to find solutions able to guarantee the advantages of robotic devices while reducing the costs [5]. Great attention has been given to handheld mechanically actuated steerable instruments. Examples are the laparoscopic instruments Maestro [6] and the LaparoFlex (TU Delft and DEAM B.V., Amsterdam, The Netherlands). Both these instruments use rigid joints in their steering mechanism to achieve bending motion in two orthogonal planes, similar to the human wrist. Rigid joints are robust and solid and therefore widely used in conventional instruments. However, when it comes to MIS, the inability to further miniaturize mechanical components due to friction limits their applications [7].

Next to the limited DOF of conventional MIS instruments, the radically different design and operation of instruments for MIS often cause ergonomic inconveniences for surgeons [8,9,10,11]. These inconveniences range from muscle fatigue and musculoskeletal pains to neural injury and worsened performance [10,12,13]. Instrument handles are the primary physical interface for the surgeon, and therefore, many studies have been dedicated to this topic [14]. One of the main conclusions of these studies points to the need for the personalization, or at the very least adaptability, of instrument handles [10,15], since it is impossible to create one handle design that suits every possible hand. Due to the high manufacturing costs associated with the conventional manufacturing of personalized products, this has long been out of reach.

### 1.3. Additive Manufacturing for Surgical Devices

Additive manufacturing (AM) or 3D printing provides new opportunities to change the design paradigm of medical devices. AM allows a 3D model to be directly converted from a Computer-Aided Design (CAD) into an object built with a layer-by-layer process. The possibility of producing complex shapes allows the number of components to be drastically reduced, in addition to increasing the functionality of the entire medical instrument. Examples of 3D-printed medical devices are the continuum robots presented by Kim et al. [16] and the 2-DOF steerable grasper DragonFlex created by Jelinek et al. [3]. A comprehensive overview of 3D-printed surgical instruments has been published previously by our group [17]. AM allows for the possibility of using different approaches, such as non-assembly 3D-printed mechanisms [18,19] or 3D-printed compliant solutions [20], which already have been successfully applied in prosthetics [21] and surgical forceps [22]. In addition, AM enables the production of personalized items at no extra cost [23,24]. Ranganathan et al. [25] 3D-printed customized forceps handles based on eight anthropometric hand parameters of Indian males. González et al. [26,27] presented and tested the design of an ergonomic pistol-grip handle that was customized to the surgeon’s specific hand size. They concluded that the use of their ergonomic handle reduced muscle fatigue and improved the ease of use of the instrument. Similarly, Sánchez-Margallo et al. [28] compared customized 3D-printed handles with standard handles and reported that the customized handles aided the surgeon’s hand–eye coordination and led to shorter execution times.

### 1.4. Objective and Requirements

**Figure 2:**
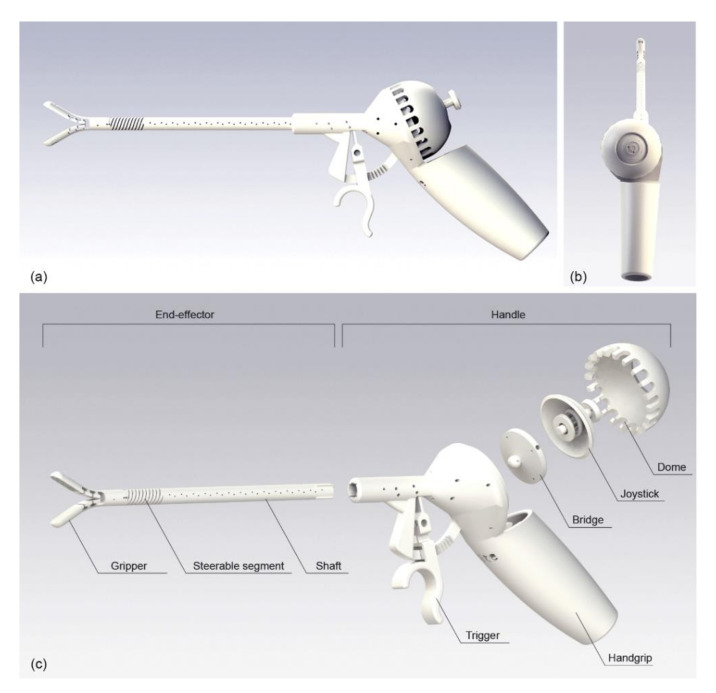
Design of 3D-GriP. (**a**) Side view; (**b**) Back view; (**c**) Exploded view with the names of the parts indicated.

In this work, we propose a design of a handheld 2-DOF cable-driven steerable instrument for MIS that maximizes the advantages of AM by making use of non-assembly design principles. The new steerable instrument (Figure 2), which we called 3D-GriP, is mechanically actuated by means of cables and was designed for AM to minimize the number of assembly steps. In order to comply with the constraints of the laparoscopic environment, it has a maximum omnidirectional bending angle of the end-effector between 40 and 60 degrees [5,29], an opening angle of the gripper of 60 degrees [30], and a shaft diameter of 8 mm [30,31,32]. The surgical instrument allows for single-hand control of both the grasping and steering mechanisms, while the design of the handle is based on ergonomic guidelines and can be customized to specific hand sizes due to the use of AM. We used Solidworks as CAD software to design the 3D-GriP.

## 2. 3D-GriP Design

### 2.1. Gripper Design

For the design of our instrument, compliant joints are used wherever possible in favor of rigid body joints. The advantage of compliant joints is that they do not suffer from the problems with clearances that occur in non-assembly rigid body joints [18,19]. An additional advantage is that compliant joints can be produced as a single, monolithic part without assembly and therefore can reduce the number of components and assembly steps. Moreover, by using flexible components to achieve motion, friction between sliding elements within the joint can be eliminated [33,34,35,36].

The compliant gripper was designed in such a way that in the relaxed position, it is in a fully open configuration with a 60-degree opening angle. This way, the forces applied on the actuation cables to close the gripper are directly translated to a (gripping) force on the tissue and thus can be easily controlled by the surgeon. Using a half-open position, as proposed by Lassooij et al. [34], has the advantage of reducing the stress on the compliant beam during operation. However, the half-open position is less convenient, as it requires the surgeon to move the gripper both to grasp and to release the tissue. In the fully open configuration, the jaws will passively return to their initial open configuration after the control input is seized due to the compliant flexures, reducing the number of active movements for the surgeon.

**Figure 3:**
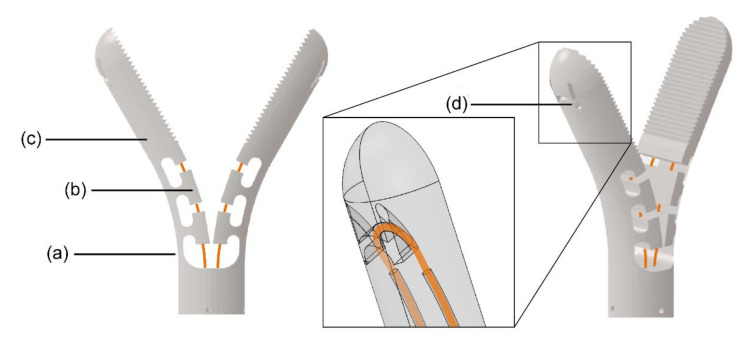
Gripper. (**a**) Compliant beam, (**b**) T-shape guiding section for the actuation cables, (**c**) jaw, (**d**) cable fixation point with its close-up made transparent. Teeth have been removed for clarity.

The design of the gripper joint was based on a compliant beam, as shown in Figure 3a, and it consists of two gripper jaws with a closed diameter of 8 mm and length of 20 mm. The tooth profile has a height of 0.5 mm and a tooth angle of 60 degrees, similar to those seen in commercial grippers for MIS [37]. The compliant beam has a thickness of 0.8 mm, in order to minimize the bending stress, while the width was kept as large as possible, to provide torsional and lateral stability. The closing of the gripper is actuated by two ∅° 0.25 mm actuation cables, which loop around the distal end of the gripper (Figure 3d) to avoid gluing or soldering on the jaws. T-shaped guiding sections were added to the compliant beams in order to gently guide the actuation cables through the joint without affecting their bendability; see Figure 3b.

### 2.2. Steerable Segment Design

**Figure 4:**
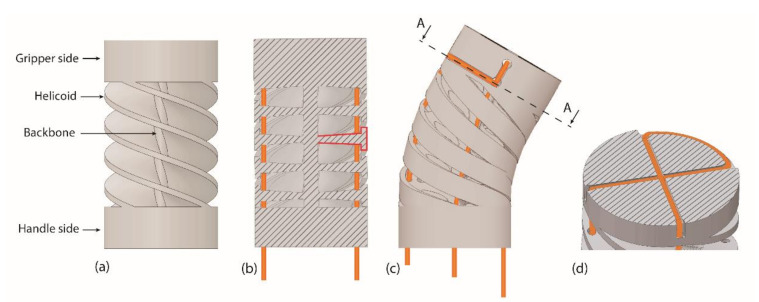
Steerable segment design. (**a**) Central backbone with four helicoids evenly placed around. (**b**) Cross-section of the segments showing the T-shape of the helicoids, highlighted in red. Cables are shown in orange. (**c**) Due to the T-shape, the helicoids touch each other at the inner curve of the segment, limiting the bending angle. (**d**) Cross-section A-A shows the cable fixation point with the looped cables.

In order to equip the instrument with two additional DOF, a steerable segment was integrated into the shaft of the device. The steerable segment used in this study was designed to achieve high torsional and axial stiffness, to avoid misalignment between the gripper and the shaft, whilst keeping a low bending stiffness to guarantee easy maneuverability. A detailed description of the design of the steerable segment is given by Culmone et al. [38]; however, for the clarity of this work, a summary follows. The steerable segment combines a compliant continuous central backbone for high axial stiffness with four helicoids evenly placed around the centerline for high torsional stiffness, homogenously distributed (Figure 4a). The helicoids have a T-shaped cross-section (Figure 4b). The T-shaped cross-section is thinnest close to the backbone and increases in thickness toward the outer side of the segment. This guarantees a low bending stiffness while at the same time limiting the maximum bending angle, which prevents failure due to excessive bending.

Two cables are used for steering, which loop around the top of the steerable segment and back down to the shaft (Figure 4c). A cross-shaped groove in the top of the segment was used to fixate the cables in place by means of friction to avoid soldering or gluing (Figure 4d). The 20 mm long steerable segment was printed as one part with the rigid shaft, in which dedicated channels guide the steering and gripper cables toward the handle.

### 2.3. Handgrip Ergonomics

A pistol-grip is used for the main shape of the handle (Figure 2), which is the preferred design for complex or multifunctional instruments [9,10,12,39]. The handle is specifically designed for right-handed use: the asymmetric grip allows for a straight alignment of the thumb and the wrist during steering, which increases the user’s comfort (Figure 2b) [40,41]. It can easily be converted to left-handed use by mirroring the design [10]. The handgrip has a bulbous shape that follows the shape of the hand [10,26] without any specific finger grooves for positioning the fingers [42], since the latter limits the positions in which it can be held. Changing the dimensions of the handle length, width, and size of the trigger allows for customization to different hand sizes.

### 2.4. Steering Control

**Figure 5:**
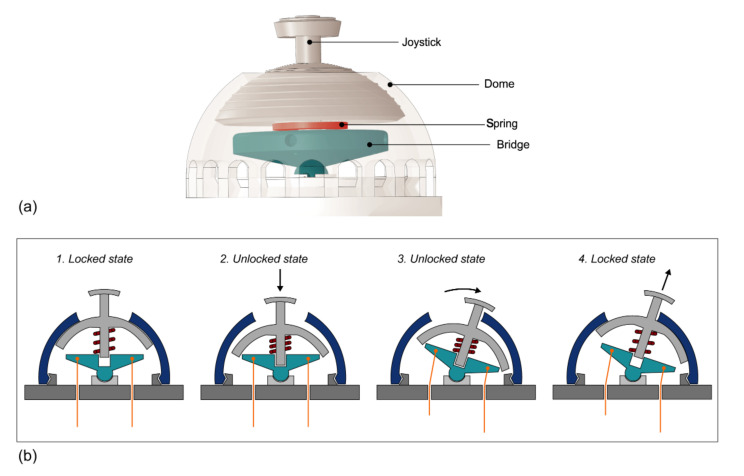
Working principle of the steering mechanism. (**a**) Three-dimensional (3D) render of the assembled joystick, showing the joystick, the dome (transparent to show the underlying components), bridge (blue), and spring (red). (**b**) Schematic drawing of the working principle of the friction lock showing the bridge (blue), spring (red), and cables (orange). (1)–(4) show the actions taken to move and lock the steerable segment.

A joystick is used for the steering system, which is actuated by the thumb. In the field of steerable surgical instruments, thumb actuation allows for more precise control in terms of motion, accuracy, and the perception of steering [40,41,43,44]. The steering mechanism itself consists of the joystick with an integrated spring, which is connected to a ball and socket joint and covered by a dome. The top part of the ball and socket joint, the bridge (Figure 5a), is the point of fixation for the cables. Rotating the joystick pulls and releases the actuation cables to steer the end-effector. In order to lock the steerable segment into any angle, an active friction lock mechanism was implemented. When no normal pressure is applied to the joystick, it is held in place by the friction between the joystick and the dome, which is caused by pre-tension in the spring (Figure 5b). This pre-tension is generated during the assembly when the dome is placed over the joystick mechanism and snapped into place. By applying pressure to the joystick, the friction lock is released, allowing for steering of the end-effector. Releasing the joystick will automatically lock the steerable segment into any angle up to 60 degrees.

The rotation of the thumb when steering, and therefore the rotation of the joystick, should be smaller than 45° in order to retain an ergonomic position [45]. Considering the desired steering angle for the end-effector of 60°, the rotation angle of the joystick cannot be transferred in a 1:1 ratio to the end-effector. Therefore, an amplification of the joystick rotation was implemented within the steering control system. To achieve this amplification, the cables’ radial distance toward the centerline of the bridge was designed to be three times larger than the cables’ radial distance toward the end-effector’s centerline. The result of this is that when rotating the joystick 20° in one direction, the end-effector will bend 60°.

### 2.5. Gripper Control

**Figure 6:**
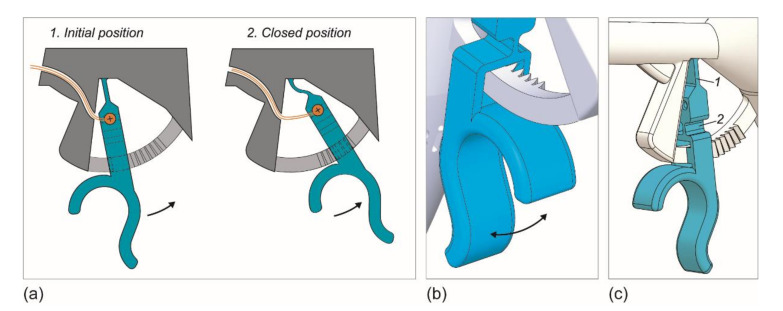
Working principle of the trigger mechanism, the trigger and flexures are highlighted in blue, the cables are shown in orange. (**a**) 1. In the initial position of the trigger, the ratchet is not locked, and the gripper is opened. 2. By moving the trigger towards the palm, the gripper closes, and the trigger is locked when the ratchet teeth are engaged. (**b**) Close-up of the internal ratchet-teeth in the locked position. (**c**) Render of the trigger indicating the two orthogonal bending flexures.

The grasping motion of the gripper is driven by a trigger, which is actuated by the index and/or middle finger (Figure 6) [44]. The trigger is automatically locked in position by means of a ratchet mechanism. In order to release the ratchet lock, the trigger needs to be moved sideways until the teeth disengage. Two orthogonal bending flexures were designed to allow the trigger to move in these two required directions (Figure 6c). As a result of the compliancy of the bending flexures, the trigger moves automatically back to the initial position when released, opening the compliant gripper. This means that only active movement for the surgeon is required to close the gripper. The actuation cables were fixated in the rigid part between the bending flexures. Although it is common in laparoscopic instruments to place the trigger mechanism inside the handle, in this case, we opted to place it outside the handle to be able to produce it with as few assembly steps as possible.

### 2.6. Prototype Fabrication and Assembly

The instrument was manufactured using a commercially available Form 3B (Formlabs, Somerville, MA, USA) 3D printer, which uses stereolithography (SLA) technology. SLA is a process in which a light source, typically a laser, hardens a liquid photopolymer in layers [46]. The handgrip, dome, bridge, and end-effector were printed using the Durable FLDCL02 resin (Formlabs, Somerville, MA, USA), exploiting the high elongation properties of the material for the compliant joints. The total volume for the parts printed with the Durable resin was 132 mL. The joystick with incorporated spring was printed with the Tough 1500 FLTO1501 resin (Formlabs, Somerville, MA, USA), with a total volume of 8.85 mL, due to its ability to produce parts that spring back under loading cycles. All parts were printed with a 50 μm layer height. The print time for the parts in Durable was 43 h, whereas the joystick was printed in 3 h and 45 min. After printing, isopropanol alcohol was used to remove the uncured resin from the prototype. Only the joystick was cured for 60 min at 70 °C in the curing chamber to enhance the spring back properties of this component.

The final prototype consists of five 3D-printed parts: (1) the end-effector, (2) handgrip with trigger, (3) bridge, (4) joystick with spring, and (5) dome. The end-effector and the handgrip could not be printed as one part because they exceed the printer’s build volume. Therefore, they were separated into two pieces and connected by a form-fit closure. The channels for the actuation cables run along the entire length of the shaft and handgrip. In order to be able to remove excess material from inside these channels, we added small drainage holes of 0.1 mm in diameter at regular intervals along the shaft (Figure 2). Furthermore, the shaft and the handgrip were printed with the long axis of the cable holes parallel to the vertical z-axis of the printer. This orientation proved best to keep the cable channels open along their entire length. The joystick and the spring were consolidated so that they could be 3D printed as one part. However, this configuration made it difficult to remove the standard support material generated by the Formlabs software. Therefore, we created custom support pillars between the coils of the spring that could be easily removed after printing.

**Figure 7:**
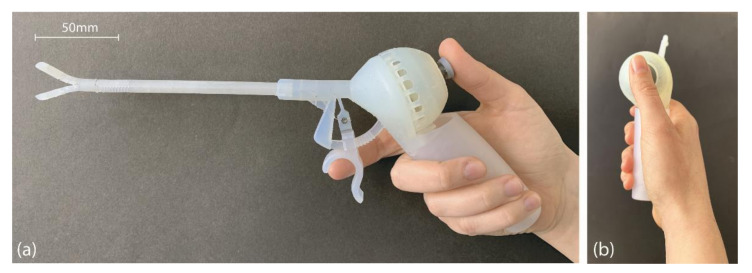
The assembled prototype. (**a**) Front view, (**b**) side view that shows the alignment of the wrist and the thumb.

To assemble the prototype (Figure 7), first, the shaft was coupled with the handgrip using the form-fit connection. To actuate the steerable segment, we used stainless steel cables (∅° 0.30 mm). The four ends of the cables were fixed using dog point screws into dedicated grooves of the bridge. Before fixation, the cables were straightened by means of weights. The gripper jaws are actuated with nitinol wires (∅° 0.25 mm) because, due to their high rigidity, they can be used to close the jaws and help open them. These wires were fixed to the trigger by means of dog point screws. After insertion and fixation of the cables, the joystick and dome were snapped into place.

## 3. Experimental Methods and Results

To assess the functionalities in steering and grasping, we performed three different measurements. First, we verified the maximum bending angle of the end-effector in four main directions, with different external loads applied to the end-effector. Second, we evaluated the required steering force applied by the user on the joystick when different external loads were applied to the end-effector, simulating steering in a surgical setting. Third, we evaluated the grasping force of the gripper on artificial tissue in relation to the required force applied by the user on the trigger.

### 3.1. Bending Angle Measurements

**Figure 8:**
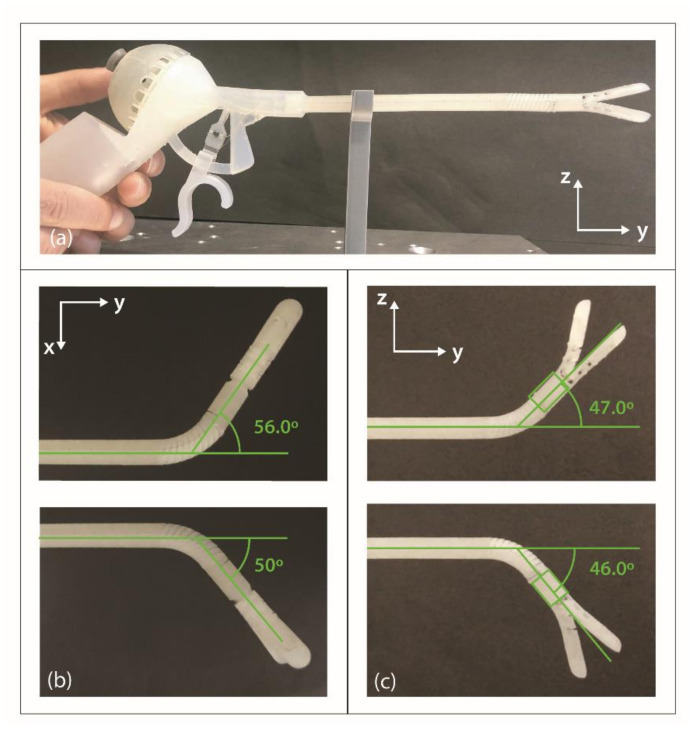
Lateral bending evaluation. (**a**) Set-up. (**b**) Lateral left/right bending in the horizontal xy-plane without load and (**c**) in the vertical yz-plane without load.

We analyzed the maximum bending angle of the end-effector by steering the joystick to its maximum position in the four main directions: upward and downward in the vertical yz-plane and right and left in the horizontal xy-plane. We repeated the measurement three times for each plane. The end-effector was able to reach an angle of approximately ±50° in both directions in the xy-plane (Figure 8b) and ±45° in the yz-plane (Figure 8c), which is somewhat lower than the desired ±60°. The video (Video S1) attached in the Supplementary Materials illustrates the omnidirectionality and the smoothness of the motion.

In addition, we evaluated the effect of different external loads on the bending performance by attaching different weights to the end of the steerable segment. Three load conditions were tested: (1) 5 g, (2) 10 g, and (3) 20 g. The load was suspended from the distal end of the steerable segment in order to only test its effects on the steerable segment and not the compliant gripper. Only the bending angle in the yz-plane was evaluated, since the direction of the load does not influence bending in the xy-plane. To measure the bending angle, we moved the joystick to its maximum up- and downward position and repeated this three times for each load condition. It was found that an increase in load decreased the bending angle in the upwards direction: 7.1% for 5 g, 19.8% for 10 g, and 28.5% for 20 g (Table 1). No considerable differences in the average of the maximum bending angle (0–2%) were observed when the steerable segment was steered downwards, regardless of the applied load.

### 3.2. Steering Force Test

#### 3.2.1. Method

**Figure 9:**
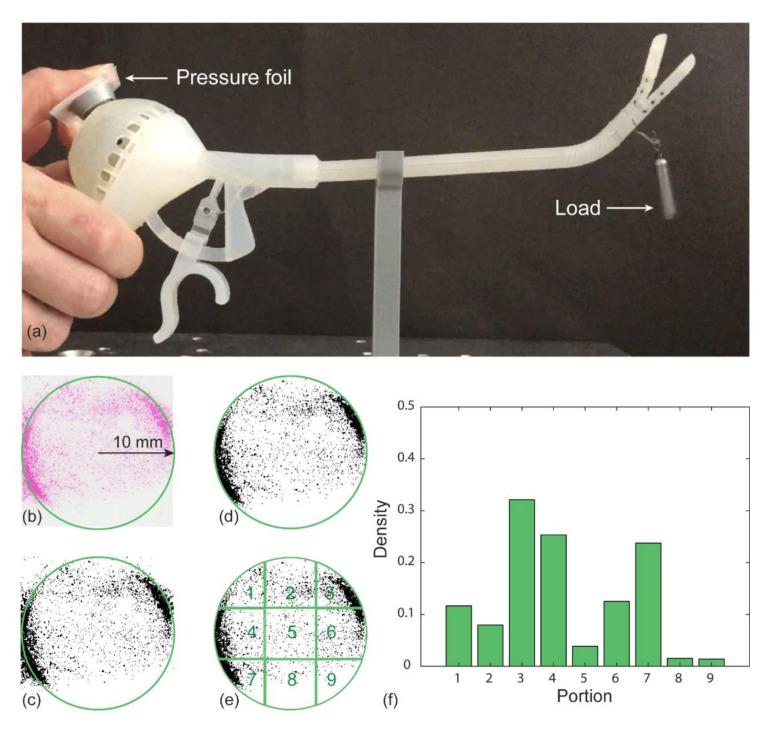
Method of force analysis on the joystick during steering. The example shown in this figure is the first repetition at the no load condition. (**a**) Set-up of the steering force measurements. (**b**) Scan of the imprinted pressure foil. The green circle represents the joystick area, the pink color shows the pressure distribution. (**c**) Black-and-white conversion of the scanned foil. (**d**) Applied mask used to analyze the circular area corresponding to the joystick area. (**e**) Segmentation of the pressure foil into nine portions. (**f**) Density of the black pixels per portion for the pressure foil for the no load condition.

In a surgical procedure, it is often necessary to move or stretch the gripped tissue. Therefore, we evaluated the force necessary to operate the joystick with the thumb in relation to the effect of different loads on the steerable segment. For this test, we applied again a load to the end of the steerable segment and moved the joystick in the four main directions (upward, downward, left, and right), after which we registered the force required for these four movements combined. We tested the following load conditions: (1) no load, (2) 5 g, (3) 10 g, and (4) 20 g. The force required to operate the joystick was measured by placing a piece of pressure foil with a sensitivity of 0.05 MPa (4LW Fujifilm Prescale, ALTHEN BV Sensors & Control, Leidschendam, The Netherlands) between the fingertip and the joystick (Figure 9a). The foil changes color when pressure is applied in a specific location. The pressure foil can be used to calculate the applied force by analyzing the density of the colored pixels. Using the pressure chart as provided by the manufacturer of the foil, the pressure value corresponding to the density can be determined. In order to calculate the total force on the joystick, the pressure is multiplied by the surface area of the joystick head.

During the test, the instrument was placed on a support that constrained the base of the handle, kept the shaft in straight position, and left the end-effector free to move. The test was performed by one of the authors and repeated three times per load condition. Although the joystick has a circular flat head with a diameter of 20 mm, we used a square piece of foil for the joystick analysis to avoid false imprints while placing and removing the foil during the test. Only the circular area of the pressure foil corresponding to the joystick head was analyzed. The acquired imprints on the pressure foil were digitalized using a calibrated scanner (Canonscan LiDE 110, Canon Netherlands N.V., ‘s-Hertogenbosch, The Netherlands) and analyzed using MATLAB R2020a (The MathWorks, Inc., Natick, MA, USA) in order to find the corresponding density. We translated the images into black and white, with a threshold of 0.8, as used in previous studies [47] where the black pixels represented the colored locations (Figure 9b,c). Then, the images were masked with a circle with the same diameter as the joystick head (Figure 9d). The digitalized figures were divided into nine portions to analyze the force distribution on the joystick, indicating on which part of the joystick the most pressure was applied by the user; see Figure 9e,f. The average black-pixel density over the three repetitions was calculated per portion and normalized for the total number of pixels.

#### 3.2.2. Results

**Figure 10:**
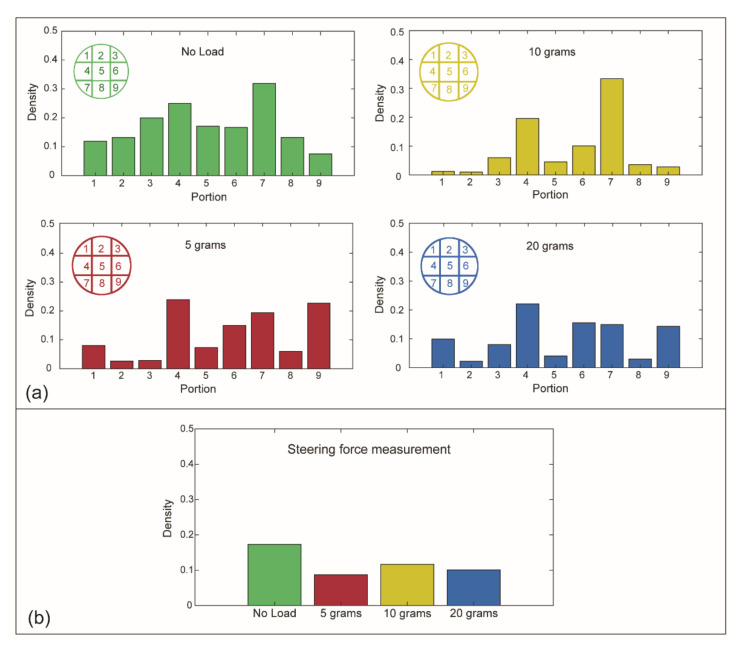
Steering force measurement. (**a**) Average of the black-pixel density per portion for each tested condition: no load (green), 5 g (red), 10 g (yellow), and 20 g (blue) applied load. (**b**) Average of the black-pixel density for each load condition on the entire area of the pressure foil.

Figure 10a shows the pressure concentration per portion for the different load conditions. For all load conditions, the black-pixel density peaks on the edges of the flat head, especially in the top right and bottom left corner (portions 4 and 7), whereas in the central vertical portions (portions 2, 5 and 8), the applied pressure reaches the lowest value.

Subsequently, we analyzed the density of the colored pixels for the entire measured area for the different load conditions (Figure 10b). The plot shows that there are no significant differences in black-pixel density (D) when increasing the load: D_0steer_ = 0.17 ± 0.05, D_5steer_ = 0.09 ± 0.04, D_10steer_ = 0.12 ± 0.03, D_20steer_ = 0.10 ± 0.02. Based on the density of the black pixels and the known surface area of the joystick head, we calculated the applied force using the pressure chart given by the manufacturer. The applied force was between 12.5 and 23.5 N, considering the 10 mm radius of the joystick head. These results indicate that the user does not need to increase the applied force to steer the joystick when the load increases in the measured range.

### 3.3. Grasping Force Test

#### 3.3.1. Method

**Figure 11:**
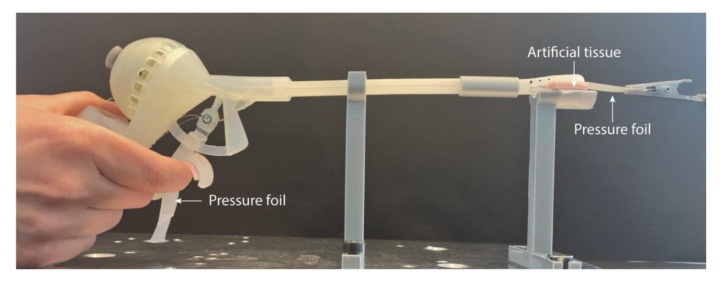
Set-up of the grasping force measurement.

Another important aspect during surgery is the force applied by the user in relation to the force at the gripper used to grasp the tissue; i.e., the efficiency of the instrument. To evaluate the grasping functionality in different scenarios, we tested the prototype on artificial silicon-based tissue (DOTFOX Snc, Siena, Italy) with three different thicknesses: 1–2 mm, 2–3 mm, and 3–4 mm. We used a set-up similar to the one used for the steering force measurement (Figure 11). Since the pressure foil is one-sided and the grasping force on both jaws of the gripper is identical when gripping, we decoupled the cables actuating the lower jaw of the gripper from the trigger and fixed the lower jaw onto customized support to prevent it from moving. Moreover, we placed a rigid tube around the steerable segment to prevent bending and analyze only the grasping functionality. We placed the artificial tissue and a piece of pressure foil with a minimum sensitivity of 0.006 MPa (5LW Fujifilm Prescale) between the jaws. The forces exerted by the user on the trigger were also measured using the same type of pressure foil. To digitalize and analyze the acquired imprints, we used the same methodology as for the imprints of the steering test described in Section 3.2.1.

#### 3.3.2. Results

**Figure 12:**
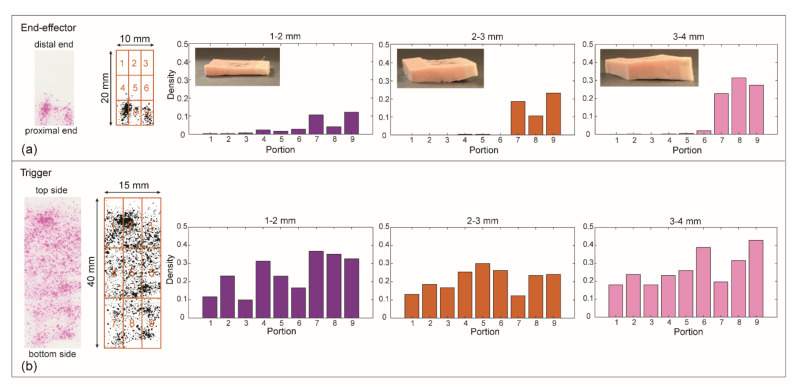
Grasping force measurements. From left to right: example of a scan of the imprinted pressure foil for 2–3 mm condition; the segmentation of the corresponding scanned foil converted into a black-and-white image; average of the black-pixel density per portion for each tested condition: 1–2 mm (purple), 2–3 mm (orange), and 3–4 mm (pink) tissue thickness. (**a**) Gripper results. (**b**) Trigger results.

The imprints for the gripper show that the pressure was concentrated at the proximal side, close to the steerable segment (portions 7, 8, and 9). For the trigger, the pressure was equally distributed among all the portions with a slightly smaller concentration on the top part of the trigger (portions 1 and 3); see Figure 12.

**Figure 13:**
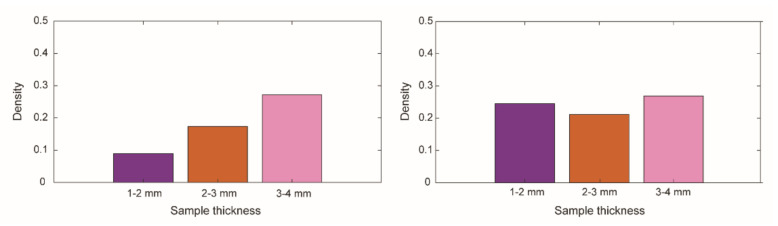
Average black-pixel concentration for the grasping force measurements. Left, the average of the black-pixel concentration of the gripper considering portions 7, 8, and 9. Right, the black-pixel concentration of the trigger for each tested tissue thickness: 1–2 mm (purple), 2–3 mm (orange), and 3–4 mm (pink).

Using the average of the black-pixel density and the surface area of the gripper and the trigger, we calculated the applied force using the pressure chart given by the manufacturer. To calculate the total force exerted by the gripper, we only used portions 7, 8, and 9, since the black-pixel density was close to zero for the gripper on the top and central parts. Therefore, the surface area of the other portions was not included in the calculation to obtain a more realistic value. The average black-pixel density (D) for portions 7, 8, and 9 was D_1–2gripper_ = 0.04 ± 0.01 for 1–2 mm, D_2–3gripper_ = 0.06 ± 0.01 for 2–3 mm, and D_3–4gripper_ = 0.09 ± 0.01 for 3–4 mm tissue thickness. The average black-pixel density for the trigger was D_1–2hand_ = 0.24 ± 0.001 for 1–2 mm, D_2–3hand_ = 0.21 ± 0.03 mm for 2–3 mm, and D_3–4hand_ = 0.27 ± 0.07 for 3–4 mm tissue thickness (Figure 13). Based on these values, the force generated by the gripper on the tissue samples was between 1 and 4.4 N, and the force applied by the user on the trigger was between 10.8 and 13.2 N. We calculated that the mechanical efficiency, and therefore the efficiency of our instrument, ranges between 10% and 30%.

## 4. Discussion

### 4.1. Production and Customization

3D-GriP was designed for use as a disposable instrument; therefore, the production process must be as fast and simple as possible. A non-assembly design can save time and costs for the total production process. In our design, the trigger mechanism is completely non-assembly, due to the use of compliant joints. For the fastest route to the total assembly of the instrument, we decided to produce the joystick mechanism out of three separate parts, which gave us access to remove supports and excess material, and place the cables through the instrument. The separate parts can be positioned easily and snapped into place. The solutions that we used for the fastest and simplest assembly can be summarized as the following design rules: (1) make use of compliant joints to create monolithic parts; (2) consolidate parts where possible; (3) ‘expose’ moving parts to ensure the material can be drained and supports removed; (4) when an assembly is unavoidable, make use of smart solutions such as snap-fit connections for quick and easy assembly.

Being 3D printed, the instrument can be customized to the patient, the procedure, and the needs of the surgeon, for instance, by changing the gripper into a needle holder or fenestrated grasping forceps. Customization of the handle depending on the surgeon’s hand size is also possible. Although we took care in our handle to adhere to ergonomic principles, it is impossible to design one handle that fits all. On the other hand, it is not necessary to change the entire design for each surgeon, since the main functionality remains the same. We addressed the customization by enabling certain specific dimensions to be easily adjustable. For instance, the length and width of the handle can be adjusted to the palm size of the surgeon, and the distance of the trigger to the handgrip can be adjusted to the length of the index finger. In addition, by mirroring the trigger design, it can be changed from right- to left-handed. For future implementation, we envision surgeons recording some of their relevant hand measurements in a personal portfolio, which can be easily implemented in the CAD design and 3D printed on demand to create surgeon-specific instruments.

### 4.2. Performance and Improvements

The low bending stiffness of the steerable segment reduces the forces required for steering and increases the ease of maneuverability. In the steering force test, we found that the force required for steering the end-effector is between 12.5 and 23.5 N, with the maximum applied force measured in no load condition. This result is counter-intuitive, but since this was the first condition tested, it might be explained by the user’s inexperience, which led to an excessive force being applied. In fact, excluding the no-load condition, the applied force ranges between 12.5 and 15.7 N. The maximum measured force applied by the user on the trigger to operate the gripper was 13.2 N. This force is comparable to the measured forces as applied by the surgeon while using conventional instruments in a laparoscopic setting that varies between 9 and 15 N for gentle pinch [42,48,49]. No data are available on the force required to steer the end-effector on commercially available steerable handheld laparoscopic instruments with fully mechanical actuation. In a future study, it would be interesting to perform a test to compare the results of the 3D-Grip to commercially available laparoscopic devices, which are especially related to the steering force.

An important aspect related to the surgeon’s comfort during laparoscopic surgery is the handle-to-tip force ratio [50]. In the grasping force test, we measured the grasping efficiency as the control-force-to-gripper-force ratio. We found that the efficiency ranges between 10% and 30%. This efficiency is comparable to common laparoscopic graspers [51]. However, this efficiency should still be improved: a higher force transmission ratio has been associated with lower muscle fatigue in the forearm, which improves the surgeon’s comfort [50], higher force feedback [52], as well as prevention of tissue slipping from the gripper, which improves performance [49]. A possible reason for a low force transmission ratio is the friction of the cables in the cable channels. Since the cable channels are difficult to clean after printing, the leftover resin may remain in the channels, which cannot be easily removed or cured. Additional drainage holes and more thorough cleaning could aid in this respect.

Using the pressure foil, we were also able to evaluate the pressure distribution on the joystick, trigger, and gripper jaws. The imprints of the joystick showed that the pressure concentration was higher on the edges, especially on the top/right and bottom/left. This result seems to indicate that more force is required for steering in the up- and downwards direction compared to the left/right direction, which can be attributed to the applied load. More research is needed to indicate whether a more equal pressure distribution can be obtained with for instance a customized joystick head. For the trigger imprints, the concentration was equally distributed over all the portions. The imprints of the gripper showed a pressure concentration of the forces at the proximal end of the jaws, which caused localized pinch force on the tissue. The limited areas of the imprints in the other portions of the gripper were too small to evaluate using the pressure foil, considering the supplier’s guidelines. To quantitatively evaluate this pressure, a possible solution would be using a pressure sensor on the gripper such as the one used by Jin et al. [53]. An interesting option to obtain a uniform distribution of the gripper forces would be an adaptable gripper such as the one proposed by Sun et al. [22].

### 4.3. Limitations and Future Studies

The verification of 3D-GriP showed that it functions comparably to existing laparoscopic instruments; however, there are some limitations to the design and tests described in this article. Since our instrument was designed for disposable use, we did not carry out a dedicated fatigue test for the compliant joints. However, we observed the compliant joint behavior and durability after repeated use of the prototype in both steering and grasping during the tests. The prototype did not experience any sign of fatigue or breakage; however, more testing is required to determine the joint fatigue and optimal dimensions for the compliant joints.

For the fixation of the cables, we initially used thread inserts and dog point screws. However, after executing the tests, the metal thread inserts in the trigger tore the material apart. In future instruments, we will experiment with alternative methods of fixating the cables, for instance by applying a small amount of the same photopolymer resin used to print the instrument at the fixation point and letting it cure. The advantage of this method is that it is quicker to apply than the thread inserts and screws, and it requires fewer parts and materials. Testing should find out whether this fixation will hold up in repeated use.

The design of the pistol-grip handle is based on well-documented ergonomic principles. However, we did not perform any tests with users to verify its comfort level. We suggest that future tests require multiple participants, preferably surgeons, as they are familiar with laparoscopic instruments, with an equal number of instruments customized to their specific hand sizes in order to verify its potential as an ergonomic instrument.

The 3D printer used for our design was a Form 3B, which is based on SLA technology and optimized for biocompatible materials. However, biocompatible materials that also have the possibility to be sterilized with different technologies, such as autoclave or gamma radiation, are limited. Moreover, since we found that the available biocompatible materials were too brittle for use in the compliant flexures, we decided to print our prototype using non-biocompatible materials to analyze the functionality of our design. In the future, we hope that new biocompatible and sterilizable materials will become available with characteristics similar to the materials we used in this study for truly biocompatible 3D printed surgical instruments.

## 5. Conclusions

In this work, we have proposed a design of a handheld steerable instrument for MIS that can be fully 3D printed. The new steerable instrument, called 3D-GriP, is fully mechanically actuated using cables. It complies with standard specifications for laparoscopic instruments, such as an omnidirectional bending between 40 and 60 degrees, a gripper opening of 60 degrees, and a shaft diameter of 8 mm. We designed a handle for the instrument based on ergonomic principles, which allows for single-hand control of both grasping and steering. Using AM allows personalizing the handle to the surgeon’s preference by adjusting specific dimensions in the CAD model. This flexibility allows the production of customized handles to increase the surgeon’s comfort. In addition, the use of AM enables a minimal part count by making use of compliant joints and snap-fit connectors. We tested the required forces to steer and operate the instrument by measuring both the input actuation force and the output grasping force. The results show that the operating forces on the handle remain below 15 N for both steering and grasping, resulting in a grasping efficiency of 10–30% for the force transmission. Although the instrument was developed for laparoscopy, our design can be easily adapted to other fields of minimally invasive surgery.

## Figures and Tables

**Table 1:** Vertical bending evaluation with different loads applied. Vertical bending in the yz-plane with no load, 5 g, 10 g, and 20 g.

	No Load (Degrees)		5 g (Degrees)		10 g (Degrees)		20 g (Degrees)	
	Upward	Downward	Upward	Downward	Upward	Downward	Upward	Downward
rep. 1	47.0	46.0	42.3	51.2	40.4	49.7	37.0	45.5
rep. 2	46.2	48.7	43.7	50.3	35.8	52.2	34.3	50.8
rep. 3	46.5	53.4	43.9	50.6	36.1	49.5	28.7	52.3
Aver.	46.6 ± 0.3	49.4 ± 3.8	43.3 ± 0.9	50.7 ± 0.5	37.4 ± 2.5	50.4 ± 1.5	33.3 ± 4.3	49.5 ± 3.5

rep. = repetition, Aver. = average.

## Data Availability

The data presented in this study are openly available in 4TU repository at https://data.4tu.nl/account/articles/16930327 (accessed on 16 December 2021).

## Supplementary Materials

The following are available online at https://www.mdpi.com/article/10.3390/ma14247910/s1, Video S1: 3D-GriP motion and tests.
